# Ecological Momentary Assessment as a Measure of Intervention Change: Evaluation in 4 Digital Mental Health Trials

**DOI:** 10.2196/69297

**Published:** 2025-09-09

**Authors:** Christian A Webb, Lori M Hilt, Caroline M Swords, Daniel M Bolt, Hadar Fisher, Simon B Goldberg

**Affiliations:** 1 McLean Hospital Belmont, MA United States; 2 Harvard Medical School Boston, MA United States; 3 Department of Psychology Lawrence University Appleton, WI United States; 4 Center for Healthy Minds and Department of Counseling Psychology University of Wisconsin–Madison Madison, WI United States; 5 Department of Educational Psychology University of Wisconsin-Madison Madison, WI United States

**Keywords:** ecological momentary assessment, rumination, mindfulness, apps, reliability

## Abstract

**Background:**

Ecological momentary assessment (EMA) is increasingly being incorporated into intervention studies to acquire a more fine-grained and ecologically valid assessment of change. The added utility of including relatively burdensome EMA measures in a clinical trial hinges on several psychometric assumptions, including that these measure are (1) reliable, (2) related to but not redundant with conventional self-report measures (convergent and discriminant validity), (3) sensitive to intervention-related change, and (4) associated with a clinically relevant criterion of improvement (criterion validity) above conventional self-report measures (incremental validity).

**Objective:**

This study aimed to evaluate the reliability, validity, and sensitivity to change of conventional self-report versus EMA measures of rumination improvement.

**Methods:**

We conducted a secondary analysis of data from 4 trials of app-based meditation interventions (N=412). Participants included adolescents (samples 1-2; n=232, 56.3%; aged 12-15 years), college students (sample 3; n=88, 21.4%; aged 18-21 years), and adults (sample 4; n=92, 22.3%; aged 18-80 years). All participants completed validated conventional self-report rumination measures (Children’s Response Styles Questionnaire, Response Styles Questionnaire, or Perseverative Thinking Questionnaire) at baseline and the postintervention time point and EMA rumination assessments throughout 3- to 4-week intervention periods (mean compliance 72.2%-80.2%). We examined the reliability of conventional self-report and EMA measures, computed correlations between measurement approaches, compared rumination improvement using standardized effect sizes, and tested criterion validity by predicting depression symptom improvement.

**Results:**

Reliability of conventional self-report rumination was high at baseline (Cronbach α=0.89-0.94) and the postintervention time point (Cronbach α=0.90-0.95) but decreased for residualized change scores (ρ= 0.71-0.90). EMA rumination showed high mean-level reliability (ρ=0.89-0.96) but substantially lower reliability for change scores (ρ=0.50-0.77). Conventional self-report and EMA measures showed medium correlations at individual time points (*r*=0.28-0.47; *P*<.01 in all cases) but nonsignificant correlations between change scores (Child and Adolescent Research in Emotion samples: *r*=0.03 and *P*=.66; Healthy Minds Program sample: *r*=0.20 and *P*=.06). Conventional self-report detected larger intervention effects than EMA measures (Cohen *d*=0.37 vs 0.14 for group differences; Cohen *d*=0.77 vs 0.17 for pretest-posttest change). Despite modest intercorrelations, both measurement approaches demonstrated incremental predictive validity for depression improvement. In the Child and Adolescent Research in Emotion samples, improvements in both conventional rumination (*b*=0.04; SE 0.017; *P*=.03) and EMA rumination (*b*=0.03; SE 0.014; *P*=.04) significantly predicted reduced depression symptoms until the 12-week follow-up. Similarly, in the Healthy Minds Program sample, both conventional rumination change (*b*=0.23; SE 0.060; *P*<.001) and EMA rumination change (*b*=20.49; SE 9.94; *P*=.04) predicted depression improvement.

**Conclusions:**

Conventional self-report and EMA measures of rumination provide distinct and clinically meaningful information. When deciding to use EMA in intervention studies, researchers should carefully consider the psychometric properties of their measures and the precise construct they intend to capture.

## Introduction

### Background

An increasing number of intervention studies (particularly mobile mental health trials) are incorporating smartphone-delivered ecological momentary assessment (EMA) to measure symptom improvement [1-9]. Proponents of EMA argue that the reliance on commonly used retrospective self-report questionnaires may be convenient and cost-effective, but these measures often lack ecological validity and are contaminated by recall bias [2,10,11]. Specifically, in contrast to EMA surveys, which are deployed in daily life and ask about current or recent (eg, “since the last survey”) experiences, conventional self-report symptom questionnaires typically ask participants to recall and summarize their average levels of symptoms over a relatively lengthy period (eg, several weeks). For example, the commonly used Beck Depression Inventory [12] and Center for Epidemiologic Studies Depression Scale [13] ask participants to recall and summarize their experience of depressive symptoms over the previous 2 weeks and 1 week, respectively. It is worth noting that common clinician-administered symptom measures (eg, Hamilton Depression Rating Scale [14] or Children’s Depression Rating Scale [15]) are not immune to these issues as they are still based on participant self-report to the interviewer and ask about time frames that extend into the past. However, skillful clinician questioning may improve participant recall and reduce memory biases relative to conventional self-report questionnaires. At an even grosser level of temporal abstraction, trait or global self-report measures ask respondents to report on their *typical* tendencies or personal characteristics without specifying a recall time frame. For example, the Response Styles Questionnaire (RSQ) [16] and Children’s RSQ (CRSQ) [17] are commonly used to assess depressive rumination in adults and children, respectively. These measures ask participants what they “generally” or “usually” do when feeling sad.

Previous research reveals that retrospective reports are often biased. For example, studies show that both children [18,19] and adults [20-24] overestimate levels of symptoms on retrospective self-report measures relative to averaged EMA reports (for evidence of age moderating this effect, see the work by Neubauer et al [25] and Zurbriggen et al [26]). Studies have also shown that, when individuals report on their experiences (eg, pain during a medical procedure) via retrospective self-report, they often incorporate information about the most intense (peak) moment and how the experience ended (ie, the so-called peak-end bias) rather than equally weighting time points and simply averaging their momentary experiences over the entire reporting time frame [27-30]. This “memory-experience gap” is due to the fact that momentary EMA requires introspection of current states (eg, emotions, thoughts, and behaviors in the here and now), whereas retrospective self-report taps into autobiographical (episodic) memory, which is limited by the extent to which past experiences and events have been accurately encoded and consolidated in memory [11,31,32]. Moreover, memory retrieval has been shown to be biased by one’s current state (eg, mood-congruent memory bias [33]). This is further complicated by the fact that individuals may shift from episodic memory to semantic memory (or a combination of the 2) when recall time frames are longer or remembering relevant details of past episodes becomes challenging [11,34]. Semantic knowledge includes more abstract beliefs or generalization about the self that are not tied to a specific time and place (eg, “I’m generally a happy person” or “I’m a worrier” [11,34,35]). One study indicated that the shift from relying on episodic memory in reporting past emotional well-being to drawing on both semantic and episodic retrieval strategies was in the 3- to 7-week range [36] (but see also the work by Walentynowicz et al [37]).

Collectively, these findings suggest that intervention researchers may benefit from relying less on convenient but often biased retrospective self-report measures and, instead, adopting EMA to measure symptom improvement. However, EMA does not necessarily provide an inherently more reliable and valid measure of intervention-related change. The repeated assessments involved in EMA protocols may introduce unique biases and careless responding (eg, random or stereotyped responding) among at least some participants [38]. For example, there is some evidence of an “initial elevation bias” (ie, an upward bias in scores on initial self-reports) when subjective reports are collected repeatedly, and this effect is more pronounced for negative mental states and physical symptoms than for positive states and behaviors [39]. However, a subsequent study found these effects to be inconsistent and minimal [40]. As another example, a recent study applied drift diffusion modeling to reaction time data from EMA affect ratings and found evidence of a shift over time in the cognitive processes that underlie survey responses. Specifically, the authors found pronounced changes in 2 central drift diffusion parameters across the repeated EMA affect ratings: increasing “drift rate” (reflecting faster processing of affective information over time) and decreased “boundary separation” (reflecting a decrease in how thoroughly participants process items and how cautiously they respond over time) [41,42]. In summary, at this stage, given conflicting findings, it is not entirely clear to what extent EMAs are more reliable and valid measures relative to conventional retrospective self-report instruments.

### Objectives

The added utility of including relatively burdensome EMA measures of change in an intervention study hinges on several psychometric assumptions, including that these measure are (1) reliable, (2) related to but not redundant with conventional self-report measures of the same construct (convergent and discriminant validity), (3) sensitive to intervention-related change, and (4) associated with a clinically relevant criterion of patient improvement (criterion validity) above and beyond conventional self-report measures (incremental validity). In this study, we included data from 4 clinical trials (N=412) of app-delivered meditation training that incorporated both EMA and conventional self-report measures of improvement in rumination. We compared these measures with regard to their reliability, validity, and sensitivity to detecting change to help inform which measures to include in future intervention research.

It is important to note that we focused on EMA measures of improvement in rumination levels over time in this study. Beyond measuring change, EMA offers unique opportunities to examine psychological dynamics that may be markers of mental health and intervention effects and are impossible to capture using traditional self-report measures. Dynamic applications of EMA have gained considerable attention in recent years, with growing interest in network models (eg, examining how symptoms influence each other over time) [43], complex dynamic system approaches (eg, studying emotional inertia and variability as indicators of psychological flexibility) [44,45], and temporal mediation analyses examining how changes in putative mediators precede and predict subsequent changes in outcomes within individuals over time [46]. However, poor reliability in EMA can obscure and interfere with the ability to detect such dynamics as well, ultimately undermining the very advantages that make EMA valuable [47].

## Methods

### Participants

Two of the trials involved adolescent samples aged 12 to 15 years selected from the community for moderate to high levels of rumination. The 80 participants in sample 1 (mean age 14.01, SD 0.99 years; n=36, 45% girls; n=69, 86% White; n=3, 4% Hispanic; median household income US $100,000-$125,000) were recruited from the Appleton, Wisconsin, area in 2018 to 2019 for a single-arm trial investigating a mindfulness mobile app. In total, 88% (70/80) of the participants completed the 3-week trial (for more details, see the study by Hilt and Swords [1]). The 152 participants in sample 2 (mean age 13.71, SD 0.89 years; n=89, 58.6% girls; n=125, 82.2% White; n=16, 10.5% Hispanic; median household income US $90,000-$100,000) were recruited in 2019 to 2020 from the same area for a randomized controlled trial investigating a mindfulness mobile app. A total of 89.5% (136/152) of the participants completed the trial (for more details, see the study by Hilt et al [48]).

Sample 3 included 88 first-year undergraduate students aged 18 to 21 years (mean age 18.51, SD 0.64 years; n=58, 66% female; n=59, 67% White; n=10, 11% Hispanic; income data not collected) recruited in 2018 from Lawrence University in Appleton, Wisconsin, for a randomized controlled trial investigating a mindfulness mobile app. They were not oversampled for any characteristic, such as elevated rumination. A total of 90% (79/88) of the participants completed the trial (for more details, see the study by Hilt et al [49]).

Sample 4 included 92 adults from the Madison, Wisconsin, area with elevated levels of depression or anxiety (ie, Patient-Reported Outcomes Measurement Information System [PROMIS] depression or anxiety T-scores of >55 [50,51]) aged 18 to 80 years (mean age 31.28, SD 13.12 years; n=75, 82% female; n=64, 70% White; n=8, 9% Hispanic; median household income US $45,000, IQR US $25,000 to US $125,000) recruited in 2021 for a randomized trial investigating a meditation-based mobile app. In total, 97% (89/92) completed the trial (more details have been provided [52]).

Power analyses for sample 1 [1], sample 2 [48], and sample 3 [49] have been previously reported. Sample 4 (unpublished) was designed to test the feasibility and acceptability of manipulating practice dosage. It was not powered to detect a particular effect. The preregistration can be found on the internet [52,53].

### Ethical Considerations

All adult participants completed informed consent procedures approved by the Institutional Review Board at Lawrence University (samples 1-3; IRBs #51418, #72518, and #8616) or the University of Wisconsin–Madison (sample 4; IRB #2019-1578; including agreement that deidentified data from the study could be shared publicly for the latter sample), and the respective institutional review boards approved each study. Given that samples 1 and 2 included minors, parents or guardians completed informed consent procedures, and youth provided assent. Data for all samples were deidentified before data processing and analysis. With regard to compensation, sample 1 received up to US $90 (US $30 at baseline and US $15 at the postintervention time point plus up to US $15 for app compliance and US $15 at each of the 2 follow-up periods). Sample 2 received up to US $105 (US $30 at baseline and US $25 at the postintervention time point plus up to US $15 for app compliance and US $15 at each of the 2 follow-up periods). Sample 3 received up to US $30 (US $5 for each assessment, US $10 for the intervention period, and up to a US $5 bonus for app use). For sample 4, participants were paid up to US $170 for completing all study activities.

### Procedure

Adolescent participants (samples 1 and 2) were recruited through letters sent to parents in the local school district and via word of mouth. They were invited to participate if a 2-item phone screen for rumination determined that they reported responding to sadness or stress with rumination at least sometimes. At baseline, adolescents and parents completed self-report questionnaires, set up the mindfulness mobile app (Child and Adolescent Research in Emotion [CARE] app) on the adolescent’s mobile device, and learned how to use it. In sample 1, all participants used the same version of the app, which prompted participants to report on mood and rumination and included a mindfulness intervention. In sample 2, adolescents were randomly assigned to use either a mood monitoring control version of the app or the mindfulness condition of the app that sample 1 used (see the Conditions section). For 3 weeks, adolescents in both samples were prompted to use the CARE app 3 times a day by notifications sent through the app. After the intervention period, participants completed online follow-up questionnaires that included the same measure of trait rumination completed as part of their baseline questionnaires.

College students (sample 3) were recruited from introductory classes, at events for first-year students, and using flyers. Participants were eligible to take part if they did not report serious suicidal concerns. Participants completed baseline questionnaires that included a measure of trait rumination and were randomized to either the mindfulness version of the CARE app or the mood monitoring control condition at baseline. During the 3-week intervention period, participants were prompted to use the app 3 times a day by notifications sent to their phone. At the postintervention time point, participants completed questionnaires that included the same measure of trait rumination completed at baseline.

Adults with elevated depression or anxiety (sample 4) were recruited through flyers placed in the community and recruitment emails sent to faculty, staff, and students at the University of Wisconsin–Madison. Participants were eligible if they were aged ≥18 years; had access to a smartphone capable of downloading the Healthy Minds Program (HMP) app; self-reported willingness to complete EMA for 4 weeks; were able to speak, read, and write in English; and had clinically elevated PROMIS depression or anxiety T-scores (>55). Exclusion criteria included previous meditation retreat experience, a regular meditation practice (weekly practice for >1 year or daily practice within the previous 6 months), previous practice under the instruction of a meditation teacher, severe depression symptoms (PROMIS depression T-score of >70), or a positive screen for alcohol dependence on the Alcohol Use Disorders Identification Test [54].

### Conditions

#### Mood Monitoring Control Condition

Samples 2 and 3 used a mood monitoring control condition through the CARE app. Participants assigned to the mood monitoring control condition were prompted to report on state mood and rumination 3 times a day without the chance of receiving a mindfulness intervention. After receiving a notification to use the app, participants reported on their current mood (ie, sad, anxious, happy, and calm) and state rumination on a scale from 0 (*not at all*) to 100 (*extremely*). The work by Hilt et al [48] provides more information.

#### Meditation Condition

Samples 1, 2, and 3 used a mindfulness meditation condition through the CARE app. Participants assigned to the mindfulness condition answered the same mood and rumination questions as those assigned to the mood monitoring control condition, and they also received mindfulness exercises. To prevent participants from learning which responses would result in receiving a mindfulness exercise, there was a 67% probability of receiving an exercise each time the app was used. If participants indicated high levels of anxiety or sadness (ie, ≥90 on the 0-100 scale), their chances of receiving a mindfulness exercise increased to 85%. If prompted to receive a mindfulness exercise, participants could select how much time they had to complete the exercise (ie, approximately 1, 5, or 10 minutes), and an exercise was randomly assigned within those parameters. After completing the mindfulness exercise, participants reported again on their current mood and rumination. The work by Hilt et al [48] provides more details.

Sample 4 used the 4-week Foundations program from the HMP mobile app. HMP includes meditation practices linked with 4 dimensions of well-being: awareness, connection, insight, and purpose [55]. Briefly, awareness practices aim to cultivate mindfulness and attention regulation, connection practices aim to cultivate healthy relationships with oneself and others, insight practices aim to cultivate an understanding of how our internal experiences (eg, emotions and thoughts) shape our wellbeing, and purpose practices aim to cultivate a connection with one’s values and a sense of meaning in daily life. The works by Goldberg et al [56] and Hirshberg et al [57] provide more details. Within this study, participants were randomly assigned to use HMP for either 5 or 15 minutes per day (defined as low- and high-dose conditions, respectively). In addition to using HMP, participants were sent EMAs assessing various aspects of well-being (awareness, connection, insight, and purpose dimensions), psychological distress (depression and anxiety), stressor exposure, and rumination 4 times per day over the 4-week intervention period.

### Measures

#### Self-Report Depressive Symptoms

For adolescents (samples 1 and 2), depressive symptoms were assessed using the Children’s Depression Inventory (CDI) [58]. The CDI is a 27-item measure of depression adapted for children and adolescents from the Beck Depression Inventory [12]. The CDI assesses the frequency and severity of depressive symptoms over the previous 2 weeks. Participants report on depressive symptoms and their severity using a 4-point scale, with higher scores on the CDI indicating greater frequency and severity of symptoms. Previous research demonstrates that the CDI is reliable and valid in child and adolescent samples [59,60]. In sample 1, the CDI showed good reliability at baseline (Cronbach α=0.82), the postintervention time point (Cronbach α=0.86), the 6-week follow-up (Cronbach α=0.88), and the 12-week follow-up (Cronbach α=0.91). In sample 2, the CDI showed excellent reliability at baseline (Cronbach α=0.90), the postintervention time point (Cronbach α=0.92), the 6-week follow-up (Cronbach α=0.91), and the 12-week follow-up (Cronbach α=0.92).

The Beck Depression Inventory–II (BDI-II) [12] was used to assess depressive symptoms in college students (sample 3). The BDI-II is a 21-item measure that asks participants to self-report on depressive symptoms experienced in the previous 2 weeks using a 4-point scale. Higher scores on the BDI-II indicate greater symptom frequency and severity. The BDI-II has demonstrated validity and reliability in college samples [61]. The measure demonstrated excellent reliability at baseline (Cronbach α=0.94), the postintervention time point (Cronbach α=0.93), the 6-week follow-up (Cronbach α=0.88), and the 12-week follow-up (Cronbach α=0.91). For samples 1 to 3 (all of which used the same CARE app), missingness for depression measures was 0.3% (1/320) at baseline and 6.9% (22/320) at the postintervention time point.

The computer-adaptive version of the PROMIS depression scale [62] was used to assess depressive symptoms in adults with elevated depression or anxiety (sample 4). The computer-adaptive PROMIS depression scale draws on a bank of 28 items. Participants rate their experience of various depression symptoms in the previous 7 days on a 5-point scale ranging from 1 (*never*) to 5 (*always*). The measure has shown strong convergent validity with legacy measures of depression, including the BDI-II [50]. As not all participants received the same items, internal consistency could not be calculated. However, short-form versions of the PROMIS depression scale have shown excellent reliability (eg, Cronbach α=0.97 for the 8-item PROMIS depression scale [63]). Missingness was very low for sample 4—0% at baseline and 3% (3/92) at the postintervention time point.

#### Self-Report Trait Rumination

Adolescent self-reported trait rumination (samples 1 and 2) was assessed using the 13-item rumination subscale of the CRSQ [17]. For each item, participants are instructed to report on how they usually respond to feelings of sadness *or stress*. Instructions were modified to include stress in line with current conceptualizations of rumination [64]. In response to each item, participants indicate whether they respond in the way described by the item on a scale from 0 (*almost never*) to 4 (*almost always*). Higher scores indicate greater rumination. Previous research suggests that the CRSQ is reliable and valid in an adolescent sample [17]. In this study, the rumination subscale of the CRSQ demonstrated good reliability in sample 1 (Cronbach α=0.89) and excellent reliability in sample 2 (Cronbach α=0.92) at baseline. At the postintervention time point, the CRSQ showed excellent reliability in sample 1 (Cronbach α=0.90) and sample 2 (Cronbach α=0.92).

College student rumination (sample 3) was assessed using the Ruminative Response Scale (RRS) from the RSQ [65]. The RRS is a 22-item measure in which participants are asked to rate whether they generally respond as described by the item on a scale from 0 (*almost never*) to 4 (*almost always*). Higher scores indicate greater frequency and severity of rumination. Previous research in college student samples suggests that the RRS has good internal consistency and moderate test-retest reliability [66]. Similar to the CRSQ, instructions were adapted to ask participants to report on their response to sadness *or stress* [64]. In this sample, the RRS showed excellent reliability at baseline (Cronbach α=0.93) and follow-up (Cronbach α=0.94). For samples 1 to 3, missingness for rumination measures was 0% at baseline and 6.6% (21/320) at the postintervention time point.

Repetitive negative thinking in the adult sample with elevated depression or anxiety (sample 4) was assessed using the Perseverative Thinking Questionnaire (PTQ) [67]. It should be noted that repetitive negative thinking is a broader construct than rumination (not solely focused on past-oriented repetitive negative thoughts but also includes, eg, future-oriented worry and present concerns). The PTQ is a 15-item measure in which participants are asked how often they respond in a particular way on a scale from 0 (*never*) to 4 (*almost always*). Items query various forms of repetitive negative thinking (eg, “I keep thinking about the same issue all the time”). Similar to the CRSQ and RSQ measures, participants are asked to rate how they *typically* respond. Higher scores indicate greater frequency of repetitive negative thought. Internal consistency and test-retest reliability, along with convergent and predictive validity, have been established for adults [67]. In this sample, the PTQ showed excellent reliability at baseline (Cronbach α=0.94) and follow-up (Cronbach α=0.95). PTQ missingness for sample 4 was 0% at baseline and 3% (3/92) at the postintervention time point.

#### EMA Rumination

In samples 1, 2, and 3, state rumination was assessed by asking participants the following questions—“How much were you focusing on your emotions?” and “How much were you focusing on your problems?”—*just before* seeing the prompt to use the app. Participants rated the degree to which they had been ruminating as described by these questions on a scale from 0 (*not at all*) to 100 (*extremely*). These questions were created in line with previous research [68,69]. In the analyses below, we focus on the latter question (problem-focused rumination) to assess rumination given (1) some concerns about the extent to which focusing on emotions in fact assesses problematic rumination (eg, participants may direct *greater* attention to their emotions by virtue of mindfulness training) [7] (see also the study by Nolen-Hoeksema et al [64], which focused on problem-focused rumination); (2) the relatively modest correlation between problem-focused and emotion-focused rumination (*r*=0.41) in this sample, which raises questions regarding internal consistency; and (3) the fact that these 2 items have yielded different patterns of findings in previous work [7]. Multimedia Appendix 1 [70] provides additional analyses based on the emotion-focused item. Participants in samples 1 to 3 had a mean survey compliance of 74% (46.71/63; SD 14.52), 79% (50.02/63; SD 14.24), and 72% (45.45/63; SD 17.78) during the 3-week intervention period, respectively. There was a nonsignificant trend for week-to-week decreases in EMA compliance (β=−0.043; *P*=.10). Multimedia Appendix 1 provides analyses showing no significant differences in EMA compliance during COVID-19 lockdowns (only 1 sample included participants enrolled during COVID-19 lockdowns).

In sample 4, participants first completed a stressor exposure item that asked them to “Think about the most stressful or negative thing that happened since you completed the last survey” and then indicate how stressed they felt “at the worst point” on a scale from 1 (*not at all*) to 7 (*very much*). Rumination was then assessed using a subsequent item—“After the stressful or negative thing happened, I was dwelling on my mistakes, failures, or losses”—which was also rated on a scale from 1 (*not at all*) to 7 (*very much*). These items were also created in line with previous research [71,72]. EMA compliance was 80.2% (89.85/112; SD 20.6%) and decreased over time by 3.4 percentage points per week (*b*=-0.034; *P*<.001).

### Clinical Trial Registration

The data analytic plan for this study was not preregistered. The original trial registration for sample 2 can be found at ClinicalTrials.gov (NCT03900416). The original trial registration for sample 4 can also be found at the Open Science Framework [53] and ClinicalTrials.gov (NCT05229406).

### Analytic Strategy

#### Reliability of Self-Report and EMA Measures

Before estimating the reliability of rumination change scores, we first report the reliability (internal consistency) of conventional retrospective self-reported rumination (for brevity, we refer to the latter simply as “retrospective rumination” in the following sections) at baseline (T1) and the postintervention time point (T2). Next, to compute the reliability of residualized change in retrospective rumination, we used the following formula, which has the reliability of retrospective rumination scores at T1 (α_T1_) and T2 (α_T1_) and the squared correlation coefficient (
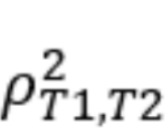
) between these 2 scores [73] as inputs:

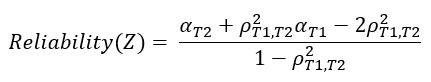



It should be noted that the reliability of residualized change (*Z*) will increase if the reliability of either T1 or T2 rumination scores increases or if the correlation between T1 and T2 rumination *decreases* (see Multimedia Appendix 1 for the reliability of raw rather than residualized change). Next, to approximate the reliability of EMA rumination, we computed a type of split-half reliability estimate evaluating the reliability of the participant mean EMA scores over time [74,75]. Specifically, we *randomly* split each participant’s vector of EMA observations into 2 subvectors, computed mean rumination in each subvector for each participant, and correlated these scores. To approximate reliability of change over time, we computed the ordinary least squares (OLS) linear slope for rumination within each subvector (ie, for each participant, we computed the slope in the 2 random subsets of EMA observations) and correlated these slopes. As each of these correlations is based on quantities derived from only half the available observations, they should be underestimates of reliability following traditional psychometric theory. As a result, a Spearman-Brown (SB) adjustment has been applied in the past [76], although some have questioned the application of this adjustment in this context [75]. Thus, in each instance, we report both the correlations from the random splits both before and following the application of the SB adjustment.

An alternative approach to evaluating the reliability of OLS slopes estimates this reliability *within* a multilevel model (MLM) that includes individual-level random intercepts and slopes (see the work by Raudenbusch and Bryk [77] for details). Consistent with the aforementioned speculation, there is evidence suggesting that reliability estimates are higher for this second approach compared to simply reporting split-half correlations without adjustment [75]. We applied MLM methods to similarly demonstrate this distinction, although we consistently report the former split-half approach both before and following SB correction to provide greater consistency to the approach taken with self-report measures and because of its frequent use in practice. Another method for evaluating change in this context would attend to individual-level empirical Bayes estimates of the slopes. Such estimates can be viewed as OLS estimates adjusted for lack of reliability. This approach was not taken in this study due to the tendency for such estimates to be “shrunken,” thus potentially introducing bias into analyses evaluating the relationships between slopes and other variables (predictor of either the slopes or outcomes) [78] (Multimedia Appendix 1).

Given that the measures of reliability for retrospective rumination (internal consistency) and EMA rumination (split-half reliability) remain somewhat different, they are not directly comparable. An important aspect of their distinction is that each attends to different sources of error variability in how the reliability of change is characterized. As noted previously, our self-report reliability estimate attends to item-related error, which is ignored (or irrelevant) in the EMA approach due to the availability of only 1 item; similarly, time-related error is ignored (or irrelevant) in the self-report approach due to a definition of change that incorporates time-related error into true change. In the analyses presented in Multimedia Appendix 1, we demonstrate (using one of our EMA datasets in which a second item could be included) how a data collection design that allows for quantification of both sources of error allows for the assessment of various reliability coefficients that could be applied to estimate reliability coefficients relevant to different conditions. Unfortunately, none of these would be applicable to our evaluation of the reliability of the OLS slope, so they are not included in our main analyses. However, we can evaluate the decrement (if any) in reliability within each measure when examining change over time.

#### Relationship Between Conventional Self-Report and EMA Measures

Similar to what was mentioned previously, before testing the association between change scores, we first tested the correlation between baseline (T1) scores on the retrospective rumination measure and mean EMA rumination over the initial 3 days of EMA. Similarly, we correlated retrospective rumination at the postintervention time point (T2) and mean EMA rumination over the previous 3 days. We selected a 3-day window for averaging in an effort to obtain a relatively representative estimate of an individual’s typical tendency to ruminate (see Multimedia Appendix 1 for analyses averaging over different numbers of days, which yielded similar findings). We also tested the association between change in retrospective rumination and EMA rumination from T1 to T2. To compute change in retrospective rumination, we saved the residuals from a model in which T2 retrospective rumination scores served as the dependent variable and T1 retrospective rumination was the predictor variable, creating a residualized change score. We focused on residualized change scores because they control for effects of regression to the mean, providing estimates of change that are independent of initial status (in addition to typically having slightly better reliability). Biostatisticians have often argued against using raw change scores for these and other reasons [79]. To compute change in EMA rumination, the slope of rumination change over the course of the trial (ie, 3 weeks or 21 days for samples 1, 2, and 3 and 4 weeks or 28 days for sample 4) was computed from participant-specific regressions of rumination scores on intervention day [74,75]. It should be noted that, as our focus in this analysis was solely on obtaining and interpreting participant-level estimates of systematic linear change over time, alternative multilevel reliability approaches were perceived as less relevant. However, the section on multilevel reliability in Multimedia Appendix 1 illustrates the application of such methods. Given the differences between samples 1 to 3 (adolescents and college students who all used the identical CARE mindfulness app for 3 weeks) and sample 4 (adults who used the HMP meditation app for 4 weeks), all analyses were conducted separately for the CARE (with sample included as a covariate in the analyses) and HMP studies (henceforth referred to as the CARE and HMP samples, respectively).

#### Comparing Rumination Improvement From Self-Report and EMA Measures

Given that the CARE sample included a mood monitoring control condition, we tested whether group differences emerged in change in rumination from T1 to T2. For retrospective rumination, we ran a regression predicting T2 rumination (adjusting for T1 rumination scores) with group as the predictor of interest. For EMA rumination, a robust linear mixed-effects model (LMM) using the statistical package *robustlmm* (version 3.1) in R (version 4.2.2; R Foundation for Statistical Computing) with a group × time interaction was specified. We specified random intercepts and slopes for participants with an unstructured covariance matrix for the random effects, allowing for correlation between random intercepts and slopes within individuals. The residual errors were assumed to have an independent covariance structure (conditional independence given the random effects). These covariance specifications are the defaults in the *robustlmm* package and were used consistently across all robust LMM analyses. A standardized effect size for the group × time interaction was estimated using the *d_growth-modeling analysis − raw_* (β_11_[time]/SD_raw_) formula recommended by Feingold [80] (for brevity, *d_growth-modeling analysis − raw_* is referred to simply as *d* in the following sections).

For the HMP sample, in which both groups had access the HMP app, we examined whether EMA rumination changed over time. To do this, we fit a robust LMM with EMA rumination as the dependent variable and time in days (ranging from 0 to 28) as the independent variable, with the nesting of observations within participants modeled using a random intercept and slope. A standardized effect size for the effect of time was estimated following the aforementioned *d_growth-modeling analysis – raw_* formula.

#### Criterion Validity of Self-Report and EMA Measures

Finally, to test whether change in traditional self-reported rumination or EMA rumination predicted improvement in depression severity (criterion), we used robust LMMs. Consistent with prior work arguing that targeting repetitive negative thinking (rumination) improves depressive symptoms [6,7,81], these analyses were an attempt to test whether improvement in either retrospective rumination or EMA rumination were related to improvement in depression. For the CARE sample, the dependent variable incorporated repeated assessment of depression over 4 time points (ie, baseline, posttreatment time point, 6-week follow-up, and 12-week follow-up) that were nested within individuals. Time was centered to represent estimated symptom scores at the final follow-up (12 weeks), and baseline depression was included as a covariate. Random intercepts and slopes were specified. To test whether either change measure (baseline to posttreatment time point) predicted improvement in depression over time (baseline to the 12-week follow-up), we included each term in an interaction with time (ie, retrospective rumination change × time and EMA rumination change × time). Each term was simultaneously included in the same model.

For the HMP sample, traditional self-reported depression was assessed only at the pre- and posttest time points. Therefore, we used OLS regression models with posttest depression as the dependent variable and pretest depression, retrospective rumination change, and EMA rumination change entered as predictors.

To reduce the influence of outliers on both the CARE and HMP samples, we winsorized any extreme values (*winsorize* function in the *DescTools* R package). Correlation and regression analyses used listwise deletion for the specific variables included in each model. Missing EMA observations within participants were handled using maximum likelihood estimation in mixed-effects models, which uses all available data and provides unbiased estimates under the missing at random assumption. Data were analyzed using R (version 4.2.2).

For correlation analyses between conventional self-report and EMA measures, our combined CARE sample (n=320) provided >80% power to detect correlations of *r*≥0.16 at an α value of .05. For the HMP sample (n=92), we had >80% power to detect correlations of *r*≥0.29 (using the *pwr* package in R). For analyses testing group differences in conventional self-reported rumination improvement in the CARE sample, we had >80% power to detect a small effect size (Cohen *f*^2^≥0.025) for between-group comparisons (*pwr* package). For mixed-effects models testing group differences in EMA rumination trajectories over time (condition × day interaction) in the CARE sample, we had >80% power to detect a small effect size of Cohen *d*≥0.25 (using the *mixedpower* package). For the HMP sample, all participants had access to the HMP app (no control group), so we tested within-subject rumination change over time. We had >80% power to detect a small to medium effect size of Cohen *d*≥0.30 for paired *t* tests of pretest-posttest rumination change (*pwr* package) and Cohen *d*≥0.22 (small effect size) for mixed-effects models testing EMA rumination decline over the study period (*mixedpower* package).

### Data Transparency Statement

Data were drawn from 4 clinical trials. Primary outcome data have been published for 3 of these trials. Primary results from the fourth trial have not yet been published. This study aggregated data across these 4 trials to evaluate the psychometric properties of EMA versus conventional self-report measures for improvement in rumination. This question has not yet been addressed within these 4 studies.

## Results

### Overview

Table 1 shows the participant demographic characteristics.

**Table 1:** Demographic characteristics of participants at baseline.

Characteristic		Sample 1 (n=80), n (%)	Sample 2 (n=152), n (%)	Sample 3 (n=88), n (%)	Sample 4 (n=92), n (%)
**Sex**					
	Male	43 (53.8)	63 (41.4)	26 (29.5)	13 (14.1)
	Female	36 (45)	89 (58.6)	58 (65.9)	75 (81.5)
	Nonbinary	0 (0)	0 (0)	4 (4.5)	4 (4.3)
	Chose not to answer	1 (1.3)	0 (0)	0 (0)	0 (0)
**Race**					
	American Indian or Alaska Native	2 (2.5)	0 (0)	0 (0)	2 (2.2)
	Asian	0 (0)	3 (2)	18 (20.5)	18 (19.6)
	Black or African American	1 (1.3)	5 (3.3)	8 (9.1)	3 (3.3)
	Multiracial	1 (1.3)	16 (10.5)	0 (0)	4 (4.3)
	Native Hawaiian or Pacific Islander	0 (0)	1 (0.7)	0 (0)	0 (0)
	White	69 (86.3)	125 (82.2)	59 (67)	64 (69.6)
	Chose not to answer	7 (8.8)	2 (1.3)	3 (3.4)	1 (1.1)
**Ethnicity**					
	Hispanic	3 (3.8)	16 (10.5)	10 (11.4)	8 (8.7)
	Non-Hispanic	75 (93.8)	136 (89.5)	78 (88.6)	83 (90.2)
	Chose not to answer	2 (2.5)	0 (0)	0 (0)	1 (1.1)

### Reliability of Self-Report and EMA Measures

#### CARE Samples

As internal consistency is computed based on the raw item scores, the 2 adolescent samples completed the CRSQ, and the other (college student) sample used the RSQ, we computed reliability separately for both measures. Reliability (internal consistency) for retrospective rumination was high at T1 (Cronbach α=0.89 for adolescents and Cronbach α=0.93 for college students) and T2 (Cronbach α=0.90 for adolescents and Cronbach α=0.94 for college students) but decreased for the residualized change score (*r*=0.71 for adolescents and 0.84 for college students). Reliability (split-half) for EMA rumination was high (*r*=0.89; SB adjusted=0.94) but decreased substantially for the change score (*r*=0.50; SB adjusted=0.67; using the reliability estimate from the MLM: *r*=0.58). There were fewer EMA time points per participant in the CARE samples (mean 50.2, SD 17.6; median 55.0) relative to the HMP sample (mean 89.9, SD 23.1; median 98). Reliability estimates are often higher when there are more time points as the slopes are estimated with less error. We recomputed reliability for the change score excluding individuals with <40 time points, which increased the reliability coefficients from *r*=0.50 (SB adjusted=0.67) to *r*=0.61 (SB adjusted=0.76).

#### HMP Sample

Reliability (internal consistency) for retrospective rumination was high at T1 (Cronbach α=0.94) and T2 (Cronbach α=0.95). The residualized change score in this sample also retained high reliability (*r*=0.90). Reliability (split-half) for EMA rumination was high (*r*=0.96; SB adjusted=0.98) but decreased for the change score (*r*=0.77; SB adjusted=0.87; it was also 0.87 using the MLM-based reliability estimate). It should be noted that the relatively high residualized change reliabilities for the HMP and CARE college student samples were due to the high reliabilities of the constituent T1 and T2 rumination scores (see the previous paragraph) and their relatively low intercorrelation (CARE college student *r*=0.66; HMP *r*=0.55 vs CARE adolescent ***r***=0.69), implying more substantial variability in real change (see, eg, formula 7.10 in the work by Crocker and Algina [82]). Multimedia Appendix 1 provides additional analyses, including convergent and divergent validity tests for EMA and the reliability of raw rather than residualized change scores that were similar.

### Correlation Between Self-Report and EMA Measures

#### CARE Samples

**Figure 1:**
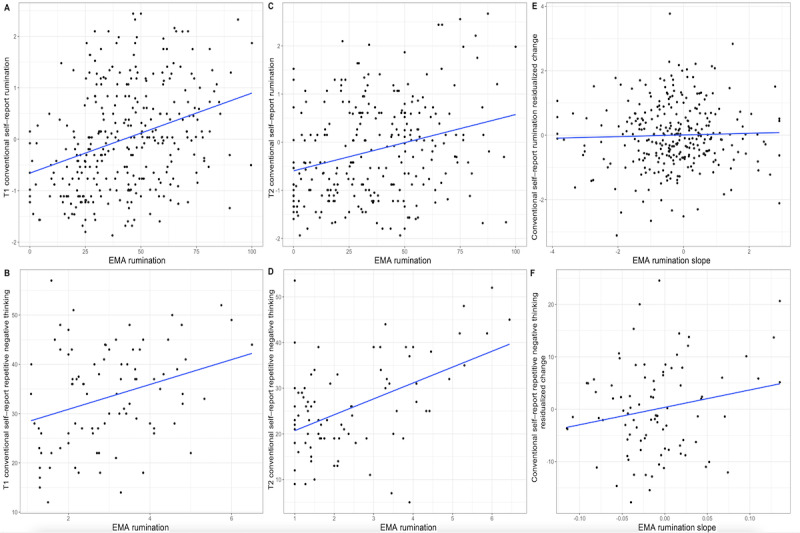
Scatterplot of the association between (1) conventional retrospective rumination at T1 and ecological momentary assessment (EMA) rumination over the subsequent 3 days for the Child and Adolescent Research in Emotion (CARE; panel A) and Healthy Minds Program (HMP; panel B) samples, (2) conventional retrospective rumination at T2 and EMA rumination over the previous 3 days for the CARE (panel C) and HMP (panel D) samples, and (3) residualized T1 to T2 change in retrospective rumination and change (slope) in EMA rumination for the CARE (panel E) and HMP (panel F) samples.

Retrospective rumination at T1 was significantly positively correlated with mean EMA rumination over the subsequent 3 days (*r*=0.35; *P*<.001; Figure 1A). Similarly, the correlation between retrospective rumination at T2 and EMA rumination over the previous 3 days was significant (*r*=0.28; *P*<.001; Figure 1C). The magnitude of these correlations is conventionally considered to be in the medium range [83].

Residualized T1 to T2 change in retrospective rumination was not significantly correlated with change (slope) in EMA rumination (*r*=0.03; *P*=.66; Figure 1E).

#### HMP Sample

Retrospective rumination at T1 was significantly positively correlated with mean EMA rumination over the first 3 days of EMA (*r*=0.31; *P*=.003; Figure 1B). Similarly, the correlation between retrospective rumination at T2 and EMA rumination over the final 3 days of EMA was significant (*r*=0.47; *P*<.001; Figure 1D). These correlations were medium to large in magnitude [83].

Residualized T1 to T2 change in retrospective repetitive negative thinking was not significantly correlated with change (slope) in EMA rumination (*r*=0.20; *P*=.06; Figure 1F).

### Comparing Rumination Improvement From Self-Report and EMA Measures

#### CARE Samples

A significant group difference emerged for change in retrospective rumination such that the mindfulness group exhibited significantly greater improvement relative to the mood monitoring control group (*d*=0.37; *b=*−0.34; SE 0.096; t_294_=−3.51; *P*<.001). In contrast, there were no group differences in EMA rumination change (*d*=0.14; *b=*−0.20; SE 0.138; *P*=.14). Overall change in the CARE trials (averaging across both conditions) was small for retrospective rumination (*d*=0.19) and EMA rumination (*d*=0.20).

#### HMP Sample

There was a significant pretest-posttest reduction in retrospective repetitive negative thinking in the HMP sample (*d*=0.77; t_88_=7.60; *P*<.001). EMA rumination decreased over time (*b*=−0.012; SE 0.0050; *P*=.02). However, the associated effect size was small (*d*=0.17).

### Predictive Validity of Self-Report and EMA Measures

#### CARE Samples

Greater pre- to postintervention improvement in both retrospective rumination (*b=*0.04; SE 0.017; *P*=.03) and EMA rumination (*b=*0.03; SE 0.014; *P*=.04) were significantly positively associated with improvement in depressive symptoms from baseline to follow-up.

#### HMP Sample

Similarly, greater pre- to postintervention improvement in both retrospective rumination (*b*=0.23; SE 0.060; *P*<.001) and EMA rumination (*b*=20.49; SE 9.94; *P*=.04) were significantly positively associated with improvement in depression symptoms from baseline to the posttest time point.

## Discussion

### Principal Findings

#### Overview

Researchers are increasingly incorporating EMA into intervention studies in an effort to obtain a more fine-grained and ecologically valid assessment of change. EMA can provide high-resolution information about treatment-relevant processes (eg, emotional, cognitive, and behavior change) while minimizing memory biases relative to conventional (retrospective) self-report measures. However, repeated EMA surveys can be burdensome to participants, which could lead to poor compliance; careless responding (eg, random or stereotyped responses to items); or a bias toward the enrollment of participants who are more motivated, thus limiting generalizability. In addition, the psychometric properties of EMA measures are often not well established before implementing them in a study (eg, researchers often create new EMA items to assess a construct of interest or adapt them from an existing self-report scale without evaluating or reporting their psychometric properties) [84]. In short, intervention researchers must carefully consider whether the benefits of implementing an EMA measure in a study outweigh potential costs and limitations. In this study, we attempted to help address the question of whether EMA is worth including by combining data from 4 trials (total N=412), all of which examined a meditation app and measured outcomes (change in rumination) via both conventional retrospective self-report questionnaires and EMA. We did so by evaluating 4 relevant criteria.

#### Criterion 1: Are EMA Measures of Rumination Reliable?

While the reliability of both retrospective rumination (at baseline and the postintervention time point) and mean EMA rumination scores was high, our findings revealed a decline in reliability when assessing change over time, especially for EMA. These results align with long-standing concerns about the unreliability of measures being compounded when measuring change [85,86]. Reliability is generally defined as the ratio of systematic (“true”) variance of interest to total observed variance. In addition to high measurement error, another reason for the low reliability in change scores is that true change variability may be low, either because of limited change or because all participants change to a nearly equivalent degree [87]. Previous EMA studies have reported relatively lower reliability for change scores [88-90]. Critically, low reliability can result in biased inferences and a loss of statistical power, leading to an inaccurate estimation of the true effect on the population [91]. Consequently, when interpreting findings, it becomes extremely challenging to determine whether the results are trustworthy or whether they are attributable to measurement error, thereby reducing the likelihood of successfully replicating findings [92]. Despite the importance of establishing reliability, many EMA studies do not adequately report the psychometrics of their measures [84].

In theory, 2 approaches to improving the reliability (and likely the validity) of EMA measures are to increase the number of time points or add more items that capture the construct of interest [93]. In this study, we relied on single-item measures of EMA rumination, which may provide less coverage of the construct of interest relative to multi-item measures [89]. However, it is important to note that increasing the number of items or assessment time points also increases the burden on participants and, consequently, may reduce compliance or increase careless responding [94]. Therefore, researchers should balance the need to improve measurement (via more items or assessment time points) with a careful consideration of participant burden. In this study, using single-item measures (a common practice in many EMA studies) also prevented us from calculating internal consistency, the method we used to compute reliability for the conventional self-report measures. Consequently, we were unable to directly compare the reliabilities of the conventional self-report and EMA measures.

Similar to increasing the number of relevant items, averaging scores across multiple measurement time points is known to improve reliability [84,93]. This approach aligns with our finding of high reliability for *mean* EMA rumination. For example, in the context of studying day-to-day changes in rumination, averaging data collected from multiple within-day occasions (as opposed to relying on a single measurement per day) to estimate rumination levels each day is expected to improve the precision of within-day rumination. In addition, the higher reliability of EMA change scores in the HMP sample relative to the CARE sample may be due to the larger number of time points collected per participant in the former sample, improving the precision of slope estimates.

In summary, the relatively low reliability of change scores (in particular for EMA measures of rumination) is concerning. It would be helpful to better understand the scope of this issue if researchers more consistently reported the reliability of change where it is evaluated given that this is not common practice yet can have a profound impact on study findings (eg, low reliability attenuating effect sizes). This can aid readers in evaluating EMA findings in a given study in light of potential psychometric limitations. The field may also benefit from additional innovative approaches to assessing the reliability of EMA measures [89].

#### Criterion 2: Correlation With Traditional Self-Report Measures

The results revealed statistically significant medium to large (*r*=0.28 to 0.47) correlations [83] between rumination assessed via conventional, retrospective self-report and the mean of EMAs. However, there were no significant correlations between changes in retrospective rumination and change in EMA rumination (*r*=0.03 to 0.20). These results are in line with those of previous studies that have reported low to moderate correlations between trait and state measures of various constructs, including anhedonia [95], affective lability [96,97], and personality traits [98,99].

Several factors may account for the relatively modest association between conventional retrospective self-report and EMA measures of rumination (especially for the change scores). First, less than perfect reliability can attenuate associations between any 2 variables. Reliability estimates were particularly low for change scores, which may explain (at least in part) the especially low correlation between those measures. Second, the distinction by Kahneman [100] between the “experiencing self” and the “remembering self” provides a useful framework for interpreting these results. According to this distinction, when participants are asked to recall their past experiences on conventional, retrospective self-report measures (in this study, asking about past ruminative thoughts), they may be especially influenced by the most intense (peak) and recent (end) levels of rumination (ie, the so-called peak-end bias) rather than equally weighting and simply averaging all time points, as reflected by the mean of repeated EMAs [27-30]. Third, EMA requires introspection of *current* states, whereas retrospective self-report measures require participants to access autobiographical (episodic) memory and provide an average report of past experiences. The accuracy of these reports is limited by the extent to which past experiences have been accurately encoded and consolidated in memory [11,31,32]. Moreover, when recall time frames are longer, individuals may shift from episodic memory to semantic memory, which includes more abstract beliefs or generalization about the self [11,34]. Thus, when asked to report on their typical tendency to ruminate on self-report measures, individuals’ responses may be influenced by relevant self-concepts (“I tend to overthink” or “I’m a worrier”), how they believe others view them, or how they would like to be perceived [101]. In summary, in addition to reduced reliability, the relatively modest correlations between conventional retrospective self-report and EMA measures of rumination may be due to the fact that the 2 measurement approaches ultimately tap into different conscious or functional “selves” [31]. As described in the following sections, the decision to include EMA or retrospective measures of change in an intervention study should be informed by which of these “selves” (experiencing vs remembering self) the researchers want to assess and expect to change.

#### Criterion 3: Sensitivity to Change

Compared to conventional retrospective self-report, EMA measures detected smaller group (intervention vs control) differences in rumination improvement (effect sizes: *d*=0.37 vs *d*=0.14 for self-report and EMA, respectively). EMA also showed less linear change in rumination over time in the intervention group (effect sizes: *d*=0.77 vs *d*=0.17 for self-report and EMA in the HMP sample, respectively). These findings have important implications for the selection of outcome measures in intervention studies. They imply that a trial may yield very different findings (eg, significant vs nonsignificant differences in outcomes between the intervention and control group) based on whether a conventional retrospective or EMA instrument was used as the outcome measure. This raises the critical question of which result is closer to the “ground truth.” The larger effect size observed for retrospective self-report could be due to this measure being a more sensitive method for capturing changes in rumination. Although speculative, EMA may be less sensitive to detecting change due the repeated nature of EMA surveys introducing unique forms of bias. For example, perhaps participants tend to align their responses with their (relatively recent) previous answers (a type of anchoring bias), potentially resulting in reduced detection of actual change. If a participant rated their rumination as a 4 on a scale from 1 to 5 on the last EMA survey, an anchoring bias may lead them to report a similar score on the next survey even if their “true” rumination score is now lower. Alternatively, EMA may provide a more *accurate* estimation of real change (which may, in fact, be modest), whereas the conventional retrospective self-report measure may overinflate estimates of change due to influences such as social desirability bias, regression to the mean, or initial elevation bias [39].

If, as discussed previously, EMA and retrospective self-reports indeed capture related but somewhat different constructs, it is possible that the pattern of results we observed is due to the intervention differentially affecting each of these facets. For example, it may be that the intervention had a larger impact on one’s episodic or semantic representations of being a ruminator (as measured via retrospective self-report) but had a more modest impact on actual moment-to-moment rumination (as measured via EMA). There may also be a causal relationship from the latter to the former. Namely, relatively modest improvements in day-to-day momentary rumination may have a proportionally large positive impact on one’s self-concept of being a ruminator.

Even if changes in retrospective self-report are biased due to the limitations of memory and there is little change in actual experience, these changes in retrospective recall may still be meaningful. Research has shown that future behaviors (eg, whether to return for a colonoscopy screening or end a romantic relationship) are *better* predicted by retrospective measures of experience than by actual momentary experiences [102]. In summary, retrospective self-reports (eg, related to one’s self-concept and tendency to engage in repetitive negative thought) may, of course, be very personally meaningful to the individual who underwent the intervention. Therefore, if the goal is to understand what individuals experienced in the moment, EMA arguably provides a more accurate measure. However, if the focus is on individuals’ global impressions of their experiences and themselves, then retrospective self-report measures may be more valuable.

#### Criterion 4: Predicting Change in Depressive Symptoms

Finally, the results of this study revealed that both retrospective and EMA measures of rumination change predicted improvement in depressive symptoms, with each measure contributing significantly beyond the effects of the other (ie, evidence of the incremental predictive validity of each measure). These findings suggest that these 2 methods assess meaningfully distinct aspects of rumination, both of which hold clinically important information. Therefore, if feasible, combining retrospective and EMA data could provide a more comprehensive picture of clinically meaningful improvement over time. It is also important to highlight that there are, of course, other reasons why a researcher may want to include EMA measures in an intervention study. For example, EMA enables the examination of symptom or emotion dynamics over time, such as changes in inertia or instability, which cannot be captured using traditional self-report methods [103]. In addition, EMA enables more sophisticated analyses of mechanisms of change, such as temporal mediation analyses that can examine how changes in putative mediators precede and predict subsequent changes in outcomes within individuals over time, addressing key limitations of traditional mediation approaches [46,104]. Nevertheless, these methodological advantages require careful attention to data quality as poor reliability in EMA can obscure the ability to detect meaningful temporal dynamics in these processes.

### Strengths and Limitations

There are several notable strengths to this study. First, we combined data across 4 trials to increase sample size (total N=412). Second, each of the trials examined a meditation app and measured outcomes via both retrospective self-report and EMA. Finally, we evaluated several metrics of reliability, validity, sensitivity to change, and incremental predictive validity to examine the value of EMA measures in an intervention study. At the same time, this study had several limitations. First, with regard to criterion 4, a well-validated clinical interview of depressive symptoms would have been preferable to a self-report instrument to serve as the “ground truth” of change during the intervention. Second, in contrast to the CARE samples, which had follow-up time points, the HMP sample analyses for criterion 4 (predicting change in depressive symptoms) were limited in testing the relationship between change in rumination and depressive symptoms over the same time frame (ie, pre- to posttreatment time point), which is problematic [105] relative to lagged analyses testing whether early change in rumination prospectively predicts subsequent depressive symptom improvement. Third, we only focused on rumination. The extent to which our findings generalize to other common outcome measures (eg, comparing conventional self-report and EMA measures of change in depression or anxiety symptoms) is unknown. Fourth, the fact that the items in the retrospective and EMA measures of rumination were not identical likely attenuated correlations. Fifth, it would have been preferable to have a multi-item rather than a single-item measure of EMA rumination, which would have allowed us to compute internal consistency and multilevel reliability estimates [89]. On the other hand, given the burden of EMA, single-item measures are *very* common, and thus, our study may generalize to the existing literature. Sixth, it may be that mindfulness training (or perhaps even repeatedly answering rumination questions in the control group) shifted how participants interpreted and responded to the rumination prompts [106-108]. For example, on the topic of the influence of meditation on item interpretation and response patterns, there is evidence of differential item functioning among meditators and nonmeditators on mindfulness measures [108]. Thus, it may be that meditation practice (eg, cultivating nonjudgmental awareness of internal experience) shifts the interpretation of mindfulness items, which could also apply to the rating of negative mental states assessed via rumination items (eg, see the previous sections for our concern about the item focusing on emotions). However, the use of only 1 item for measurement in EMA, combined with the relatively low sample sizes, precluded our ability to meaningfully evaluate longitudinal measurement invariance. Seventh, the fact that the included samples and trials differed in patient characteristics (eg, age) and methods (eg, different measures of rumination) could be considered a limitation. On the other hand, this allowed us to test the consistency of the findings (eg, reliability of and correlations between retrospective and EMA measures) across these samples and study differences (which, overall, were quite consistent, which provides some evidence, albeit limited, for the generalizability of the findings). Finally, although our samples were diverse in age and setting, they were predominantly White (59/88, 67%-69/80, 86%). This limits the generalizability of our findings across racial and cultural groups. Previous research suggests that the strength of association between rumination and psychological outcomes may differ across cultural backgrounds [109].

### Conclusions

Despite these limitations, the results of this study provide insights into the utility of conventional self-report and EMA measures of rumination that are relevant considerations for researchers when designing studies and interpreting results. First, the reliability of change in rumination over the course of the intervention was relatively low, especially for EMA. This is concerning given that change over time is precisely what intervention researchers are most interested in. Increasing the number of EMA assessment time points and using multi-item scales while being mindful of not overburdening participants may improve the precision with which change over time is estimated. The frequently observed low reliability of change is often presented as a concern about the measurement of change more generally [110]. However, beyond the reliability of measures at each time point, the most critical factor is the correlation between measures across time points, which can be viewed as an index of the variability of change. As this will be a feature of both study and population conditions that will not be consistent across studies or under the direct control of the investigator, we concur with previous commentaries on the issue that reliability of change scores should be evaluated on a case-by-case basis rather than dismissed out of hand [111-113].

Second, conventional self-report and EMA measures of rumination were modestly correlated at a given time point, with no significant correlation between changes in retrospective and EMA measures of rumination over time. Despite this, changes in both measures of rumination predicted decreased depressive symptoms. This suggests that conventional self-report and EMA measures of rumination are not redundant. Rather, each measure captures distinct and clinically meaningful facets of rumination (ie, remembered vs experienced rumination). Using both conventional self-report and EMA measures of rumination in combination, when feasible, may provide a more comprehensive picture of clinical change. It can be appreciated that the tendency for conventional self-report measures to administer many items at few time points and the tendency for EMA to administer fewer items at many time points also provide complementary designs that minimize the effects of different sources of error (in the case of self-report, item-related error and, in the case of EMA, time-related error), which may be useful to quantify using generalizability theory techniques, and that can be incorporated into a multilevel reliability analysis. There is value for future research to carefully consider such complexities related to the psychometric properties of EMA measures of rumination and, ideally, ultimately standardize measures of rumination in the EMA literature.

## Appendix

Multimedia Appendix 1 Supplementary results and tables presenting additional analyses.

## Abbreviations

BDI-II: Beck Depression Inventory–II
CARE: Child and Adolescent Research in Emotion
CDI: Children’s Depression Inventory
CRSQ: Children’s Response Styles Questionnaire
EMA: ecological momentary assessment
HMP: Healthy Minds Program
LMM: linear mixed-effects model
MLM: multilevel model
OLS: ordinary least squares
PROMIS: Patient-Reported Outcomes Measurement Information System
PTQ: Perseverative Thinking Questionnaire
RRS: Ruminative Response Scale
RSQ: Response Styles Questionnaire
SB: Spearman-Brown

## Data Availability

The code and datasets generated or analyzed for sample 4 are available in the Open Science Framework repository [114]. The datasets generated or analyzed for samples 1 to 3 are not publicly available as the institutions require a signed data sharing agreement, but are available from the corresponding author on request.
