# Supplementary material for: Ecological Momentary Assessment as a Measure of Intervention Change: Evaluation in 4 Digital Mental Health Trials
Source: J Med Internet Res. 2025 Sep 9;27:e69297. doi: 10.2196/69297 (PMC12457869; doi:10.2196/69297)
Supplement: Multimedia Appendix 1 [file jmir_v27i1e69297_app1.docx]

**Supplementary Materials**

**Supplemental Results**

***Relationship between conventional self-report and EMA measures.*** In the main text we reported the correlation baseline (T1) scores on the conventional self-reported (trait) rumination measure and mean EMA rumination over the initial 3 days of EMA (for brevity, we refer to the former as simply “retrospective rumination”). Similarly, we correlated retrospective rumination (repetitive negative thinking for the HMP sample) at post-intervention (T2) and mean EMA rumination over the prior 3 days. Here, we re-run the analyses but changing the number of days over which we average EMA rumination scores (from 3 days to 2 or 4 days). For the CARE sample, the T1 and T2 correlations were *r* = .33 and *r* = .24 when averaging EMA rumination over 2 days. The T1 and T2 correlations were *r* = .35 and *r* = .29 when averaging EMA rumination over 4 days. For the HMP sample, the T1 and T2 correlations were *r* = .31 and .45 when averaging EMA rumination over 2 days. The T1 and T2 correlations were *r* = .31 and .46 when averaging EMA rumination over 4 days.

***Convergent and divergent validity of EMA.*** For the CARE datasets, in addition to assessing rumination via EMA we also administered EMA items assessing state sadness and happiness. If the EMA measures are reliable and valid, we would expect the rumination items to correlate positively with sadness and inversely with happiness. Indeed, the mean within-person correlation (computing the bivariate correlation for each individual separately, and then averaging it across all subjects) between EMA rumination and sadness was *r =* .44 and between rumination and happiness was *r* = -.34.

For the HMP sample, in addition to assessing rumination via EMA we also administered EMA items assessing state depression (“In the last 15 min, I felt depressed”) and happiness since the last EMA assessment (“Think about the most enjoyable or happy time since you completed the last survey. At the best point, how happy did you feel?”). The mean within-person correlation between EMA rumination and depression was *r* = .41 and between rumination and happiness was *r* = -.20.

***Emotion-focused rumination.*** In response to an anonymous reviewer, for the CARE samples we also tested associations between emotion-focused rumination and conventional self-reported rumination, which yielded very similar findings relative to the results reported in the main text which focus on problem-focused rumination. Retrospective rumination at T1 was significantly positively correlated with mean EMA rumination over the subsequent 3 days (*r* = 0.36, *p* < .001). Similarly, the correlation between retrospective rumination at T2 and EMA rumination over the prior 3 days was significant (*r* = 0.27, *p* < .001). Residualized T1-T2 change in retrospective rumination was not significantly correlated with change (slope) in EMA rumination (*r* = 0.03, *p* = .573).

***Reliability of raw change in rumination.*** In the main test we report the reliability of residualized change in conventional retrospective rumination. Here we report the reliability of raw change scores using the below formula (Irwin, 1966; Stanley, 1967):

$$\rho_{D}= \frac{\alpha_{T1}\sigma_{T1}^{2}+ \alpha_{T2}\sigma_{T2}^{2}-2\rho_{T1,T2}\sigma_{T1}\sigma_{T2}}{\sigma_{T1}^{2}+ \sigma_{T2}^{2}-2\rho_{T1,T2}\sigma_{T1}\sigma_{T2}}$$

The reliability of raw change scores (D) were as follows: CARE Adolescents ($r_{D}=$.66), CARE College ($r_{D}=$.81), and HMP sample ($r_{D}=$.88).

***Assessing individual change in EMA rumination using multilevel models (MLMs) instead of subject-specific ordinary least squares (OLS) regressions.*** In the CARE sample, the correlation between slopes extracted from MLM and OLS regression slopes was very high (*r =* .90) and a similar pattern of findings emerged when substituting the MLM-derived slope for the OLS regression slopes. Specifically, split-half reliability for the MLM-derived slope was low (*r* = .62). Residualized T1-T2 change in retrospective rumination was not significantly correlated with change (MLM-derived slope) in EMA rumination (*r* = 0.07, *p* = .245). There was a non-significant trend for greater pre- to post-intervention improvement in EMA rumination (*b =* 0.04, *SE =* 0.021, *p* = .056) being positively associated with improvement in depressive symptoms from baseline though follow-up.

For the HMP sample, the correlation between MLM-derived slope and OLS regression slopes was also very high (*r* = .90) and a similar pattern of findings emerged when substituting the MLM-derived slope for the OLS regression slopes. Split-half reliability for the MLM-derived slope was acceptable (*r* = .78). Residualized T1-T2 change in retrospective rumination was not significantly correlated with change (MLM-derived slope) in EMA rumination (*r* = 0.19, *p* = .072). Greater pre- to post-intervention improvement in EMA rumination was positively associated with improvement in depression symptoms from baseline to post-test (*b* = 25.14, *p* = .033).

***Multilevel reliability.*** As noted by an anonymous reviewer, designs in which multiple item measures are collected across multiple time points allow for quantifications of different sources of error variance and a possible multilevel perspective on reliability. Such a perspective may also be a very useful one in which to compare EMA and conventional self-report measurement, as EMA typically involves many time points of measurement with fewer items per time point, while conventional self-report measurement typically involves more items at fewer time points. Using generalizability theory, quantification of these different sources of variance can even support the identification of designs that render highest reliability (e.g., that identify the optimal numbers of items per time point and time points). Our study did not pursue this approach for a couple of primary reasons: (1) the EMA designs studied did not involve multiple items (one involved two items, but the two items were viewed as measuring different constructs), and (2) the conventional self-report design involved only two timepoints, not permitting a separation of true and error time components of change. As noted in the paper, there is also an “apples versus oranges” issue at play in the use of a person slope to estimate reliability with EMA, while residualized change is used with self-report. However, we fully agree with the reviewer’s comment that this is a useful perspective to take on the data, and use (as an illustration) the EMA dataset (three CARE samples) for which we have two items per time point.

Using the mlr function in the Psych (Revelle, 2024) package in R, we can take advantage of the full crossing of the person (ID) x time point x item designs to obtain variance components (using the R routine lmer) for each of the three factors and each of the two-way interactions, allowing the three-way-interaction to function as a quantification of error (Residual) (see Tables S1 and S2). From the variance component estimates shown below, we observe approximately equal variance component estimates associated with the ID x time and ID x items components. Since the design of the EMA data involve many more time points of measurement than items (2, in this example), reliability (generalizability) suffers under any scenario under which person x item variability is viewed as the primary source of error against which true person variability is compared. As noted, for the current EMA dataset, such error reflects in large part the belief that the two items are measuring different constructs, so was not incorporated into our reliability calculations. Additionally, as some of the ID x time variability is viewed as real change in our analysis (that attributed to the linear slope of change and its variability across persons), this perspective is less aligned with the person variability of interest in the paper.

***Covid Lockdowns.*** Of the 4 samples included in our paper, only one sample (“Sample 2”) recruited participants during a time period (June 17, 2019 - September 3, 2020) that overlapped with COVID lockdown in Wisconsin (March 25 – May 13, 2020). Within Sample 2, only 6 participants were enrolled in the study during the lockdown. In other words, only 1.5% (6/412) of the total sample size included in our paper were enrolled during COVID lockdowns. The average number of study days in lockdown was 34.8% (i.e., among those 6 participants with any lockdown exposure, most of their time in the study was not during lockdown). Among this minority of participants, there were no significant differences in EMA compliance during vs. not during COVID lockdown (p-values = 0.425). Participants in Study 1 (recruited 2018-2019) and Sample 3 (recruited 2018) were recruited before the COVID lockdowns. All participants in Study 4 were enrolled after the COVID lockdown.

**References:**

William Revelle (2024). psych: Procedures for Psychological, Psychometric, and Personality Research. Northwestern University, Evanston, Illinois. R package version 2.4.3

**Table S1**

Components of variance estimated by lmer

|  | Variance | Percent |
| --- | --- | --- |
| ID | 203.79 | 0.29 |
| Time | 4.22 | 0.01 |
| Items | 4.20 | 0.01 |
| ID x Time | 122.23 | 0.17 |
| ID x Items | 128.32 | 0.18 |
| Time x Items | 0.00 | 0.00 |
| Residual | 245.27 | 0.35 |
| Total | 708.04 | 1.00 |

**Table S2**

**Multilevel Reliabilities**

| RkF | 0.98 | Reliability of average of all ratings across all items and times (Fixed time effects) |
| --- | --- | --- |
| R1R | 0.52 | Generalizability of a single time point across all items (Random time effects) |
| RkR | 0.96 | Generalizability of average time points across all items (Random time effects) |
| Rc | 0.5 | Generalizability of change (fixed time points, fixed items) |
| RkRn | 0.96 | Generalizability of between person differences averaged over time (time nested within people) |
| Rcn | 0.24 | Generalizability of within person variations averaged over items (time nested within people) |
